# Physical and mental health functioning after all-cause and diagnosis-specific sickness absence: a register-linkage follow-up study among ageing employees

**DOI:** 10.1186/s12889-017-4051-z

**Published:** 2017-01-25

**Authors:** Minna Mänty, Tea Lallukka, Jouni Lahti, Olli Pietiläinen, Mikko Laaksonen, Eero Lahelma, Ossi Rahkonen

**Affiliations:** 1 Department of Public Health, Faculty of Medicine, University of Helsinki, Tukholmankatu 8B, P.O. Box 20, FIN-00014 Helsinki, Finland; 2 Finnish Institute of Occupational Health, P.O. Box 40, FIN-00251 Helsinki, Finland; 3 Finnish Centre for Pensions, FIN-00065 Eläketurvakeskus, Helsinki, Finland

**Keywords:** Sickness absence, Health, Functioning

## Abstract

**Background:**

Sickness absence has been shown to be a risk marker for severe future health outcomes, such as disability retirement and premature death. However, it is poorly understood how all-cause and diagnosis-specific sickness absence is reflected in subsequent physical and mental health functioning over time. The aim of this study was to examine the association of all-cause and diagnosis-specific sickness absence with subsequent changes in physical and mental health functioning among ageing municipal employees.

**Methods:**

Prospective survey and register data from the Finnish Helsinki Health Study and the Social Insurance Institution of Finland were used. Register based records for medically certified all-cause and diagnostic-specific sickness absence spells (>14 consecutive calendar days) in 2004–2007 were examined in relation to subsequent physical and mental health functioning measured by Short-Form 36 questionnaire in 2007 and 2012. In total, 3079 respondents who were continuously employed over the sickness absence follow-up were included in the analyses. Repeated-measures analysis was used to examine the associations.

**Results:**

During the 3-year follow-up, 30% of the participants had at least one spell of medically certified sickness absence. All-cause sickness absence was associated with lower subsequent physical and mental health functioning in a stepwise manner: the more absence days, the poorer the subsequent physical and mental health functioning. These differences remained but narrowed slightly during the follow-up. Furthermore, the adverse association for physical health functioning was strongest among those with sickness absence due to diseases of musculoskeletal or respiratory systems, and on mental functioning among those with sickness absence due to mental disorders.

**Conclusions:**

Sickness absence showed a persistent adverse stepwise association with subsequent physical and mental health functioning. Evidence on health-related outcomes after long-term sickness absence may provide useful information for targeted interventions to promote health and workability.

## Background

In most Western countries, the workforce is ageing rapidly.[1] This highlights the importance of understanding factors contributing to health, functioning as well as workability among ageing employees. Long-term sickness absence is recognized as a useful measure of physical and mental health in studies of working populations.[2, 3] However, research in the area has concentrated primarily on the causes of sickness absence, whereas much less attention has been paid to changes in health-related outcomes after periods of sickness absence.

For the employee, sickness absence is the time for the recovery, whereas at the population level, extended sick leave has been shown to be a risk marker for severe future health outcomes, such as disability retirement [4, 5] and premature death.[3, 6] In addition, some studies have suggested that long-term sickness absence may be a prognostic marker for common chronic conditions [7] and severe injuries, such as hip fractures.[8] Thus, it is possible that sickness absence is also associated with subsequent poor physical and mental health, both important determinants of work disability [9–11] and life expectancy.[12, 13] However, few studies to date have addressed how periods of sickness absence are reflected in the subsequent course of physical and mental health over time [14, 15]. These previous studies have included employees aged 25 to 51 years, thus excluding ageing employees who are approaching their retirement age. In addition, the main focus has been on general health status and persistency of the associations between sickness absence and subsequent physical and mental health functioning has not been assessed. Moreover, the associations of diagnosis-specific sickness absence with different domains of health functioning remains unknown. Such evidence is needed to find effective ways to support employees with their return-to-work process, and to prevent further functional decline and the severe health outcomes related to long-term sickness absence.

We examined the association of all-cause and diagnosis-specific sickness absence with subsequent changes in physical and mental health functioning among ageing municipal employees.

## Methods

### Participants

**Fig. 1:**
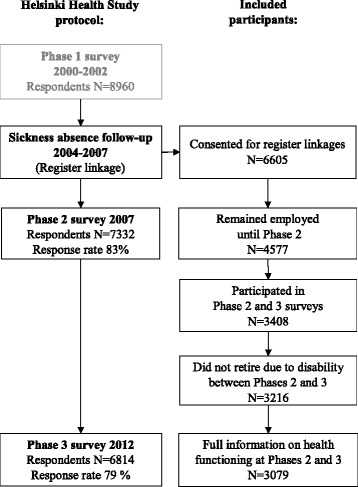
Study flow chart

This study is part of the Helsinki Health Study (HHS), which examines the health and well-being among the ageing employees of the City of Helsinki, Finland. The staff of the City of Helsinki is in charge of general local administration, health care, social welfare, education and culture, public transport and technical service, and includes hundreds of different occupations from manual workers to non-manual clericals, professionals and managers [16]. Fig. 1 shows inclusion of the participants during the different phases of the data collection. *Phase 1* data were collected by postal surveys in 2000, 2001 and 2002 among employees reaching 40, 45, 50, 55 or 60 years of age in each year (*n* = 8960) [16]. *Phase 2* follow-up survey was conducted in 2007 (*n* = 7332, response rate 83%) and *Phase 3* in 2012 (*n* = 6814, response rate 79%). From the year 2004 onwards, the survey data were linked to the Social Insurance Institution of Finland’s register on sickness absence data using unique personal identification numbers assigned to each Finnish resident. The data linkage was done for Phase 1 respondents who gave their written consent for such linkage (74%). According to the non-response analysis, the Phase 1 and follow-up data, as well as the data for linkage consenters satisfactorily represent the target population.[16, 17] However, men, younger employees and those with long sickness absence were slightly overrepresented among the non-responders and non-consenters [18]. Further details of the Helsinki Health Study and the data collection can be found in our cohort profile [16].

For the purposes of this study, we focused on those who remained employed until Phase 2 (*n* = 4577) and those who participated in Phase 2 and 3 surveys (*n* = 3408) (Fig. 1). Furthermore, we included only respondents who did not retire due to disability between Phases 2 and 3 (*n* = 3216) and those with full information on physical and mental health functioning at Phases 2 and 3 (*n* = 3079). Excluded participants tended to have poorer physical and mental health functioning at Phase 1 as compared to those who were included in the current analyses (data not shown).

### Sickness absence

In Finland, all medical certificates for sickness absences lasting longer than 10 working days at a time (≥14 consecutive calendar days) are registered at the Social Insurance Institution of Finland and include a medically confirmed diagnoses (ICD-10). For these sickness absence spells employees are entitled for reimbursement. Shorter (<14 consecutive calendar days at a time) sickness absence spells are covered by the employers. The net days of reimbursed sickness absence with their medical diagnoses were available from the year 2004 onwards and were recorded for each participant during a 3-year period prior to the return of the Phase 2 questionnaire in 2007. Parental leave and absence from work due to caring for a sick child were extracted from the sickness absence days.

The *total number of all-cause sickness absence days* during the 3-year follow-up was categorized into four groups: 1) 0–13 days, 2) 14–30 days, 3) 31–60 days, and 4) > 60 days. The first category (0–13 days) includes employees with no sickness absence days as well as those with short, non-reimbursed sickness absence spells during the follow-up. The categories 2–4 include participants with various combinations of reimbursed (and non-reimbursed) sickness absence spells. For example, one sickness absence spell of 31–60 days or multiple separate 14-day spells together sum up to 31–60 days, resulting in classifying a participant in the 3rd category. In addition to all cause sickness absence, we examined the four most common *diagnostic causes of the reimbursed sickness absence*: diseases of musculoskeletal system (M00-M99), mental disorders (F00-F99), external causes (S00-T98) and diseases of respiratory system (J00-J99). Due to small numbers, all other diagnostic groups were classified as ‘other’.

### Physical and mental health functioning

Physical and mental health functioning were measured by the physical (PCS) and mental (MCS) component summary indices of the Short-Form 36 (SF-36) health questionnaire [19] at Phases 2 and 3. The SF-36 questionnaire includes eight subscales: physical functioning, role limitations due to physical problems, bodily pain, general health perceptions, mental health, role limitations due to emotional problems, social functioning and vitality. These eight subscales were compressed into the two component summaries: the physical (PCS) and mental (MCS) component summaries. The subscales are continuous with the scores ranging from 0 to 100, with higher scores indicating better functioning. The SF-36 has a good construct validity and high internal consistency as well as test-retest reliability. As a continuous measure SF-36 is well-suited for analysing changes of functioning and it captures even small changes in functioning better than commonly used dichotomous health measures. [19]

### Covariates

Survey based information on age and gender, and register based information on occupational class were obtained at Phase 2. Occupational social class included managers (managerial and administrative work) and professionals (e.g. teachers and doctors), semi-professionals (e.g. nurses), routine non-manual employees (e.g. child minders and assistant maids), and manual workers (e.g. transport and cleaning work) [10]. Data on retirement between Phases 2 and 3 were obtained from the national registers of the Finnish Centre for Pensions, providing complete information on all retirement events.

### Statistical methods

Study population characteristics are reported as percentages and mean values with standard deviations (SD). The effect of all-cause and diagnosis-specific sickness absence was analysed in two steps: *First*, the adjusted differences in PCS and MCS scores at Phases 2 and 3 were calculated by number of sickness absence days (Table 2) and diagnostic causes (Tables 3 and 4) using linear regression analysis. To assess whether the observed group differences at the end of the sickness absence follow-up remain also during the subsequent 5-year follow-up, we report the mean differences separately for Phases 2 and 3. *Second*, adjusted group specific changes of PCS and MCS scores from Phase 2 to Phase 3 were calculated with repeated measures ANOVA using PROC MIXED procedure in SAS (Tables 1, 2 and 3). This group specific approach allows to examine whether the change in physical and mental health functioning differs between the different sickness absence groups during the subsequent follow-up. We tested potential interactions between gender and sickness absence, retirement and sickness absence, and occupational class and sickness absence on PCS and MCS scores during the follow-up. None of the interactions were statistically significant (range from *p* = .106 to *p* = .909) and the analyses were conducted in pooled data. The results are reported as regression coefficients (β) and their 95% confidence intervals (95% CI). The SAS 9.4 Statistical Package was used for all analyses (SAS institute Inc., Cary, NC, USA).

## Results

During the 3-year follow-up of sickness absence, 70% of participants had less than 14 consecutive sickness absence days, 14% had 14–30 days, 7% had 31–60 days, and 9% had more than 60 absence days (Table 1). A high number of sickness absence days was more common in women, among lower occupational classes and those who entered old-age retirement during the subsequent five-year follow-up. In addition, a high number of absence days was associated with lower subsequent PCS and MCS scores. Among those who had sickness absence spell(s) lasting at least 14 consecutive calendar days (*n* = 938), 32% had sickness absence due to musculoskeletal causes, 17% due to mental causes, 16% due to external causes, 8% due to respiratory disease(s) and 36% due to other causes (data not shown). The absentees could have spells due to different diagnoses.

**Table 1:** Baseline (2007) characteristics by sickness absence in 2004–2007 among 3079 women and men

Baseline (year 2007) variable	n (%)	Medically certified sickness absence* in 2004-2007			
		0–13 days n (%)	14–30 days n (%)	31–60 days n (%)	>60 days n (%)
	3079 (100)	2141 (70)	416 (14)	236 (7)	286 (9)
Gender					
Women	2512 (82)	1701 (79)	360 (87)	236 (87)	246 (86)
Men	567 (18)	440 (21)	56 (13)	31 (13)	40 (14)
Age, *mean (SD*)	47.1 (5.4)	46.9 (5.4)	47.3 (5.4)	47.1 (5.4)	47.9 (5.4)
Occupational class					
Managers/professionals	1067 (35)	841(39)	104 (25)	62 (26)	60 (20)
Semi-professionals	740 (24)	528 (25)	100 (24)	50 (21)	62 (22)
Routine non-manuals	935 (30)	571(27)	151 (36)	99 (42)	114 (40)
Manual workers	337 (11)	201 (9)	61 (15)	25 (11)	50 (18)
Old-age retirement between 2007 and 2012					
No	2322 (75)	1650 (77)	310 (74)	171 (73)	191 (66)
Yes	757 (25)	491 (23)	106 (26)	65 (28)	95 (33)
Health functioning					
PCS score, *mean (SD*)	49.6 (7.8)	51.0 (6.7)	48.1 (8.1)	47.1 (8.5)	43.7 (10.1)
MCS score, *mean* (*SD*)	52.2 (9.3)	52.6 (8.6)	52.2 (9.5)	51.5 (10.5)	49.7 (12.0)

NOTE Figures are numbers (percentages) unless otherwise stated

*Sickness absence lasting ≥ 14 consecutive calendar days

### Physical health functioning

Higher number of all-cause sickness absence days was associated with lower levels of subsequent physical functioning in a stepwise manner: the more absence days during the previous three years, the poorer the subsequent physical health functioning (Table 2). Those with 14–30 absence days had−2.7 (95% CI−3.5 to −2.0), those with 31–60 absence days−3.8 (95% CI−4.8 to−2.8) and those with over 60 absence days−7.1 (95% CI −8.0 to−6.2) points lower physical health functioning scores at the end of the sickness absence follow-up in 2007 as compared to those with less than 14 absence days when adjusting for gender and age. Overall, physical health functioning declined slightly (β -0.9, 95% CI −1.2 to −0.7) during the subsequent five-year follow-up from 2007 to 2012. However, as functioning tended to decline less among those with the history of higher number of sickness absence days, the observed differences between the absence groups narrowed somewhat, that is, the group differences tended to be smaller in 2012 than in 2007 (Table 2). Further adjustment for occupational class and retirement only slightly attenuated the observed associations.

**Table 2:** Adjusted Differences in Cross-sectional Means and Group Specific Longitudinal Changes (β coefficients) of SF-36 Physical (PCS) and Mental (MCS) Health Functioning Scores from 2007 to 2012 by number of sickness absence days in 2004–2007 (*n* = 3079)

Absence days in 2004–2007:	2007		2012		Change from 2007 to 2012	
	β	(95% CI)	β	(95% CI)	β	(95% CI)
PCS						
Model 1						
*All*					*−0.9*	(−*1.2 to*−*0.7*)
0–13 days*	ref.		ref.		−1.2	(−1.5 to−0.9)
14–30 days**	−2.7	(−3.5 to−2.0)	−2.5	(−3.3 to−1.6)	−0.9	(−1.7 to−0.1)
31–60 days**	−3.8	(−4.8 to−2.8)	−3.4	(−4.5 to−2.3)	−0.8	(−1.9 to 0.3)
>60 days **	−7.1	(−8.0 to−6.2)	−5.1	(−6.1 to−4.1)	0.8	(−0.3 to 2.0)
Model 2						
*All*					−*0.9*	(−*1.2 to*−*0.7*)
0–13 days	ref.		ref.		−1.2	(−1.5 to−0.9)
14–30 days	−2.5	(−3.2 to−1.7)	−2.1	(−3.0 to−1.3)	−0.9	(−1.6 to−0.1)
31–60 days	−3.5	(−4.5 to −2.5)	−3.1	(−4.1 to−2.0)	−0.8	(−1.9 to 0.3)
>60 days	−6.7	(−7.6 to−5.8)	−4.6	(−5.6 to−3.6)	0.8	(−0.3 to 1.9)
MCS						
Model 1						
*All*					*1.0*	(*0.6 to 1.3*)
0–13 days	ref.		ref.		0.7	(0.3 to 1.1)
14–30 days	−0.5	(−1.5 to 0.5)	0.1	(−0.8 to 1.0)	1.4	(0.5 to 2.3)
31–60 days	−1.2	(−2.5 to 0.1)	−0.6	(−1.8 to 0.5)	1.3	(−0.1 to 2.7)
>60 days	−3.0	(−4.1 to −1.8)	−1.6	(−2.7 to−0.5)	2.1	(0.7 to 3.6)
Model 2						
*All*					*1.0*	(*0.6 to 1.3*)
0–13 days	ref.		ref.		0.7	(0.3 to 1.1)
14–30 days	−0.7	(−1.7 to 0.3)	−0.0	(−0.9 to 0.9)	1.4	(0.5 to 2.3)
31–60 days	−1.4	(−2.6 to −0.1)	−0.8	(−2.0 to 0.4)	1.3	(−0.1 to 2.7)
>60 days	−3.2	(−4.3 to −2.0)	−1.8	(−2.9 to−0.7)	2.1	(0.7 to 3.5)

* 0 absence days or non-reimbursed sickness absence only (absence periods lasting less than 14 consecutive calendar days at a time)

**number of reimbursed sickness absence days from the absence periods lasting ≥ 14 consecutive calendar days

Model 1 adjusted for age and gender

Model 2 adjusted for Model 1 + occupational class in 2007 and old-age retirement between 2007 and 2012

Table 3 shows the results according to the main diagnostic causes of sickness absence. The associations between sickness absence and physical health functioning were the strongest for diseases of musculoskeletal and respiratory systems when adjusting for gender and age. These associations remained also during the subsequent follow-up from 2007 to 2012 and after further adjustments for occupational class and old-age retirement. (Table 3).

**Table 3:** Adjusted Differences in Cross-sectional Means and Group Specific Longitudinal Changes (β coefficients) of SF-36 Physical Health Functioning Score (PCS) from 2007 to 2012 by sickness absence diagnosis (*n* = 3079)

Diagnosis of sickness absence* in 2004–2007:	2007		2012		Change from 2007 to 2012	
	Mean	(95% CI)	Mean	95% CI	Mean	95% CI
Model 1						
Musculoskeletal disease						
*No*	ref.		ref.		−1.1	(−1.4 to−0.9)
*Yes*	−6.8	(−7.7 to−5.9)	−4.9	(−5.9 to−3.9)	0.8	(−0.3 to 1.8)
Mental disorders						
*No*	ref.		ref.		−0.9	(−1.2 to−0.6)
*Yes*	−1.2	(−2.4 to 0.1)	−1.9	(−3.2 to−0.6)	−1.7	(−3.0 to−0.4)
External causes						
*No*	ref.		ref.		−0.9	(−1.2 to−0.7)
*Yes*	−1.5	(−2.7 to−0.2)	−1.9	(−3.3 to−0.6)	−1.4	(−2.8 to 0.0)
Respiratory disease						
*No*	ref.		ref.		−0.9	(−1.2 to−0.7)
*Yes*	−3.7	(−5.4 to−1.9)	−4.6	(−6.5 to−2.7)	−1.8	(−4.2 to 0.6)
Other						
*No*	ref.		ref.		−1.0	(−1.3 to−0.8)
*Yes*	−3.1	(−3.9 to−2.2)	−2.1	(−3.0 to−1.2)	−0.0	(−0.9 to 0.9)
Model 2						
Musculoskeletal disease						
*No*	ref.		ref.		−1.1	(−1.4 to−0.9)
*Yes*	−6.2	(−7.1 to−5.3)	−4.3	(−5.2 to−3.3)	0.8	(−0.3 to 1.9)
Mental disorders						
*No*	ref.		ref.		−0.9	(−1.2 to−0.6)
*Yes*	−0.9	(−2.1 to 0.3)	−1.7	(−3.0 to−0.4)	−1.7	(−3.0 to−0.4)
External causes						
*No*	ref.		ref.		−0.9	(−1.2 to−0.7)
*Yes*	−1.2	(−2.5 to 0.0)	−1.7	(−3.0 to−0.4)	−1.3	(−2.7 to 0.0)
Respiratory disease						
*No*	ref.		ref.		−0.9	(−1.2 to−0.6)
*Yes*	−3.6	(−5.3 to−1.8)	−4.4	(−6.3 to−2.5)	−1.7	(−4.1 to 0.6)
Other						
*No*	ref.		ref.		−1.1	(−1.4 to−0.8)
*Yes*	−2.8	(−3.7 to−2.0)	−1.8	(−2.7 to−0.9)	−0.0	(−0.9 to 0.9)

NOTE. To be included as an absentee in a particular diagnostic category, participants had to have at least one new sick leave episode for that diagnosis during the 3-year exposure window. The reference group for each diagnostic category is participants with no sickness absence for that specific diagnosis

*Sickness absence lasting ≥ 14 consecutive calendar days (reimbursed sickness absence)

Model 1 adjusted for age and gender

Model 2 adjusted for Model 1 + occupational class in 2007 and old-age retirement between 2007 and 2012

### Mental health functioning

Mental health functioning was lower among those with a history of over 60 absent days (β -3.0, 95% CI −4.1 to–1.8) as compared to those with less than 14 absence days when adjusting for gender and age (Table 2). Overall, mental functioning improved slightly (β 1.0, 95% CI 0.6 to 1.3) during the subsequent five-year follow-up, and the observed association among those with a history of over 60 absent days narrowed as functioning tended to improve more over time among those with a history of higher number of sickness absence days (Table 2). Further adjustment for occupational class and retirement only slightly attenuated the observed associations.

Table 4 shows that subsequent mental health functioning was lower especially among participants who had history of sickness absence with diagnosis of mental disorders and slightly lower among those with a history of sickness absence due to other diagnoses. These associations narrowed during the subsequent follow-up but remained statistically significant also after further adjustments.

**Table 4:** Adjusted Differences in Cross-sectional Means and Group Specific Longitudinal Changes (β coefficients) of SF-36 Mental Health Functioning Score (MCS) from 2007 to 2012 by sickness absence diagnosis (*n* = 3079)

Diagnosis of sickness absence* in 2004–2007:	2007		2012		Change from 2007 to 2012	
	Mean	(95% CI)	Mean	95% CI	Mean	95% CI
Model 1						
Musculoskeletal disease						
*No*	ref.		ref.		1.1	(0.8 to 1.4)
*Yes*	1.0	(−0.1 to 2.1)	−0.2	(−1.3 to 0.8)	−0.1	(−1.3 to 1.0)
Mental disorders						
*No*	ref.		ref.		0.8	(0.4 to 1.1)
*Yes*	−7.0	(−8.5 to−5.5)	−2.8	(−4.2 to−1.4)	5.0	(2.8 to 7.3)
External causes						
*No*	ref.		ref.		0.9	(0.6 to 1.2)
*Yes*	0.0	(−1.5 to 1.6)	1.5	(0.0 to 2.9)	2.4	(0.8 to 3.9)
Respiratory disease						
*No*	ref.		ref.		1.0	(0.6 to 1.3)
*Yes*	−1.0	(−3.2 to 1.2)	−1.1	(−3.2 to 0.9)	1.0	(−1.4 to 3.3)
Other						
*No*	ref.		ref.		0.9	(0.6 to 1.3)
*Yes*	−1.3	(−2.4 to−0.3)	−1.0	(−2.1 to−0.1)	1.3	(0.2 to 2.4)
Model 2						
Musculoskeletal disease						
*No*	ref.		ref.		1.1	(0.8 to 1.4)
*Yes*	0.9	(−0.2 to 2.0)	−0.4	(−1.5 to 0.6)	−0.1	(−1.3 to 1.0)
Mental disorders						
*No*	ref.		ref.		0.8	(0.4 to 1.1)
*Yes*	−7.1	(−8.5 to−5.6)	−2.9	(−4.3 to−1.5)	5.0	(2.8 to 7.3)
External causes						
*No*	ref.		ref.		0.9	(0.6 to 1.2)
*Yes*	−0.1	(−1.6 to 1.4)	1.4	(−0.1 to 2.8)	2.3	(0.8 to 3.9)
Respiratory disease						
*No*	ref.		ref.		0.9	(0.7 to 1.3)
* Yes*	−1.1	(−3.2 to 1.0)	−1.2	(−3.2 to 0.9)	0.9	(−1.4 to 3.3)
Other						
*No*	ref.		ref.		0.9	(0.6 to 1.3)
*Yes*	−1.4	(−2.5 to−0.4)	−1.1	(−2.1 to−0.1)	1.3	(0.1 to 2.4)

NOTE. To be included as an absentee in a particular diagnostic category, participants had to have at least one new sick leave episode for that diagnosis during the 3-year exposure window. The reference group for each diagnostic category is participants with no sickness absence for that specific diagnosis

*Sickness absence lasting ≥ 14 consecutive calendar days (reimbursed sickness absence)

Model 1 adjusted for age and gender

Model 2 adjusted for Model 1 + occupational class in 2007 and old-age retirement between 2007 and 2012

## Discussion

Our study showed that all-cause sickness absence was persistently associated with lower subsequent physical and mental health functioning in a stepwise manner: the more absence days, the poorer the subsequent physical and mental health functioning. The adverse association for physical health functioning was strongest among those with sickness absence due to diseases of musculoskeletal or respiratory systems, and for mental functioning among those with sickness absence due to mental disorders.

Our findings on the adverse associations of sickness absence, and subsequent physical and mental health functioning corroborate the previous findings for younger employees and more generic health outcomes,[14, 15] and moreover, they extend the existing research by providing novel new evidence on the associations between length of sickness absence and subsequent physical and mental health functioning among ageing employees. Furthermore, previous studies have neglected the associations between diagnosis-specific sickness absence and different domains of subsequent health functioning.

Poor physical and mental health have been associated with sickness absence [9] as well as disability retirement [10, 11] and premature death [12, 13]. Thus, it is possible that secular changes in physical and mental health functioning may partly underlie the previously observed associations between high sickness absence levels and further severe health-related outcomes [3–6]. However, this topic clearly needs further investigation. Such studies are important for the prevention of premature health decline among ageing employees with a history of long-term sickness absence and may also offer insights into potentially modifiable risk factors. For example, various interventions aiming to improve health behaviours, such as diet, exercise and cognitive training, have proved efficient in promoting physical [20, 21] and mental [22, 23] health among older people. Whether such interventions could be efficient also for restoring workability and preventing further severe health decline among ageing employees with a history of long sickness absence, needs to be explored in future studies.

In our study, the observed differences in health functioning by the number of sickness absence days narrowed somewhat during the subsequent five year follow-up (Table 2). This narrowing was due to the fact that the changes in health functioning varied between the absence groups: physical functioning tended to decline less and mental health functioning tended to improve more over time among those with a history of higher number of sickness absence days (Table 2). It is possible that these observations reflect gradual improvements of the diseases and conditions behind the sickness absence spells. However, the sickness absence spells were relatively long and likely to reflect long-term health problems. Unfortunately, we are unable evaluate these associations further in our data and future studies are needed to explore this issue in more detail.

### Methodological considerations

The strengths of our study include: first, the longitudinal design which allowed us to examine the associations between sickness absence and subsequent changes in health functioning. Second, the sickness absence data were derived from complete national registers, which contributes to the validity and reliability of our measure of sickness absence. Furthermore, our measure of sickness absence allowed us to examine the effect all-cause as well as diagnosis-specific sickness absence on physical and mental health functioning. Third, to ascertain physical and mental health functioning, we used the validated SF-36 physical and mental component summary scores. Despite the observed group differences were fairly small, many of them were close to or greater than 3 points, which has been previously suggested as a clinically significant difference [24].

This study had also limitations. Firstly, when assessing the generalisability of the results, some characteristics of the data need to be considered. We studied an occupational cohort from the public sector with the majority of participants being women which limits the generalizability of the results. However, the participation was broadly similar among women and men, although slightly more women than men responded to the surveys [16]. Moreover, the gender distribution reflects that within the City of Helsinki and the Finnish municipal sector in general. Furthermore, as we restricted our analyses to those who remained employed until the end of the sickness absence follow-up and those who did not retire due to disability during the subsequent follow-up for health functioning, the data are likely to be affected by the ‘healthy worker effect’ as is typical for occupational cohorts. Our exclusions may have caused some underestimation in the observed associations as those who exit from work life thorough disability retirement are known to have poorer physical and mental health [11, 25]. We would also like to refer to our non-response and attrition analyses [16, 17] that suggest that the data are broadly representative of the target population. We are aware that our results cannot be generalised to the Finnish labour force in general, but only to the target populations of employees of the City of Helsinki and, with caution, to the Finnish municipal sector. Further studies are needed to investigate how disability retirement affects subsequent physical and mental health functioning.

Second, measures on physical and mental health functioning were based on self-reports and the possibility for under- or over-reporting cannot be ruled out. Third, the level of pre-absence health functioning is likely to be reflected also in the level of post-absence health functioning. Thus, those with initially poorer health may be more likely to have certified sickness absence, and be more likely to have worse health also after their sickness absence. However, although we study longitudinal associations we do not argue that the found associations are causal in nature. Fourth, as in most observational studies, residual and unmeasured confounding is possible. Fifth, we also acknowledge that as our measure on sickness absence is based on absence spells lasting at least 14 consecutive calendar days, the used reference category may include some participants with shorter sickness absence spells up to 14 absence days. This may cause underestimation to the observed group differences in health functioning and, as a result, the true differences between the different sickness absence categories might be larger than those observed in our study.

## Conclusions

Our study showed that sickness absence had a persistent adverse stepwise association with subsequent physical and mental health functioning among ageing employees. Evidence on the health-related outcomes after long-term sickness-absence may be useful in planning targeted interventions to promote health and workability.

## Abbreviations

CI: Confidence Interval
MCS: Mental component summary score of the Short-Form 36 Health Survey
PCS: Physical component summary score of the Short-Form 36 Health Survey
SAS: Statistical Analysis Software developed by SAS institute
SD: Standard Deviation
SF-36: Short-Form 36 Health Survey

## References

[1] Christensen K, Doblhammer G, Rau R, Vaupel JW (2009). Ageing populations: the challenges ahead. Lancet.

[2] Marmot M, Feeney A, Shipley M, North F, Syme SL (1995). Sickness absence as a measure of health status and functioning: from the UK Whitehall II study. J Epidemiol Community Health.

[3] Kivimäki M, Head J, Ferrie JE, Shipley MJ, Vahtera J, Marmot MG (2003). Sickness absence as a global measure of health : evidence from mortality in the Whitehall II prospective cohort study. Br Med J.

[4] Lund T, Kivimäki M, Labriola M, Villadsen E, Christensen KB (2008). Using administrative sickness absence data as a marker of future disability pension: the prospective DREAM study of Danish private sector employees. Occup Environ Med.

[5] Kivimäki M, Forma P, Wikström J, Halmeenmäki T, Pentti J, Elovainio M (2004). Sickness absence as a risk marker of future disability pension: the 10-town study. J Epidemiol Community Health.

[6] Vahtera J, Pentti J, Kivimäki M (2004). Sickness absence as a predictor of mortality among male and female employees. J Epidemiol Community Health.

[7] Kivimäki M, Head J, Ferrie JE (2008). Singh-Manoux a, Westerlund H, Vahtera J, et al. Sickness absence as a prognostic marker for common chronic conditions: analysis of mortality in the GAZEL study. Occup Environ Med.

[8] Stenholm S, Vahtera J, Kjeldgård L, Kivimäki M, Alexanderson K (2014). Length of sick leave as a risk marker of hip fracture: a nationwide cohort study from Sweden. Osteoporos Int.

[9] Laaksonen M, Kääriä S, Leino-Arjas P, Lahelma E (2011). Different domains of health functioning as predictors of sickness absence - a prospective cohort study. Scand J Work Env Health.

[10] Lahelma E, Martikainen P, Rahkonen O, Roos E, Saastamoinen P (2005). Occupational class inequalities across key domains of health: results from the Helsinki Health Study. Eur J Public Health.

[11] Pietiläinen O, Laaksonen M, Rahkonen O, Lahelma E (2011). Self-rated health as a predictor of disability retirement - the contribution of ill-health and working conditions. PLoS One.

[12] Cooper R, Kuh D, Hardy R (2010). Objectively measured physical capability levels and mortality: systematic review and meta-analysis. BMJ.

[13] DeSalvo K, Bloser N, Reynolds K, He J, Muntner P (2005). Mortality prediction with a single general self-rated health question. A meta-analysis. J Gen Intern Med.

[14] Gustafsson K, Marklund S (2011). Consequences of sickness presence and sickness absence on health and work ability: a Swedish prospective cohort study. Int J Occup Med Environ Health.

[15] Vahtera J, Westerlund H, Ferrie JE, Head J, Melchior M, Singh-Manoux A (2010). All-cause and diagnosis-specific sickness absence as a predictor of sustained suboptimal health: a 14-year follow-up in the GAZEL cohort. J Epidemiol Community Health.

[16] Lahelma E, Aittomäki A, Laaksonen M, Lallukka T, Martikainen P, Piha K (2012). Cohort Profile: The Helsinki Health Study. Int J Epidemiol.

[17] Laaksonen M, Aittomäki A, Lallukka T, Rahkonen O, Saastamoinen P, Silventoinen K (2008). Register-based study among employees showed small nonparticipation bias in health surveys and check-ups. J Clin Epidemiol.

[18] Martikainen P, Laaksonen M, Piha K, Lallukka T (2007). Does survey non-response bias the association between occupational social class and health?. Scand J Public Health.

[19] Ware J, Kosinski M, Keller SD, Ware JE, Kosinski M, Keller SD (1994). SF-36 Physical and Mental Component Summary Measures: A User’s Manual.

[20] Cooper R, Mishra GD, Kuh D (2011). Physical activity across adulthood and physical performance in midlife: findings from a British birth cohort. Am J Prev Med.

[21] Cooper AJM, Simmons RK, Kuh D, Brage S, Cooper R (2015). Physical Activity, Sedentary Time and Physical Capability in Early Old Age: British Birth Cohort Study. PLoS One.

[22] Rovio S, Kåreholt I, Helkala E-L, Viitanen M, Winblad B, Tuomilehto J (2005). Leisure-time physical activity at midlife and the risk of dementia and Alzheimer’s disease. Lancet Neurol.

[23] Ngandu T, Lehtisalo J, Solomon A, Levälahti E, Ahtiluoto S, Antikainen R (2015). A 2 year multidomain intervention of diet, exercise, cognitive training, and vascular risk monitoring versus control to prevent cognitive decline in at-risk elderly people (FINGER): A randomised controlled trial. Lancet.

[24] Samsa G, Edelman D, Rothman M, Williams G, Lipscomb J, Matchar D (1999). Determining clinically important differences in health status measures: a general approach with illustration to the Health Utilities Index Mark II. Pharmacoeconomics.

[25] Lahelma E, Pietiläinen O, Rahkonen O, Lallukka T, Pietilainen O, Rahkonen O (2015). Common mental disorders and cause-specific disability retirement. Occup Environ Med.

